# Keyhole Approach for Clipping Anterior Circulation Aneurysms: Clinical Outcomes and Technical Note

**DOI:** 10.3389/fsurg.2021.783557

**Published:** 2021-12-07

**Authors:** Dongqi Shao, Yu Li, Zhixiang Sun, Xintao Cai, Xialin Zheng, Zhiquan Jiang

**Affiliations:** Department of Neurosurgery, The First Affiliated Hospital of Bengbu Medical College, Bengbu, China

**Keywords:** keyhole approach, anterior circulation, aneurysm, SAH, pterional approach, supraorbital approach

## Abstract

**Purpose:** Keyhole craniotomy is a minimally invasive approach for the treatment of anterior circulation aneurysm. In this study, we evaluated the benefits and value of the keyhole approach by analyzing the surgical results in 235 patients with anterior circulation aneurysm treated by the keyhole approach and identifying lessons learned from addressing various complications in this approach.

**Patients and Methods:** This was a retrospective study in a single institution of 235 surgical patients with 248 anterior circulation aneurysms who had the supraorbital keyhole approach (SKA) or pterional keyhole approach (PKA) between January 2016 and January 2021. The modified Rankin Scale (mRS) was used to measure long-term results during follow up.

**Results:** All 235 patients' aneurysms were fully clamped and have not recurred. Among them, 31 (13.2%) had intraoperative aneurysm rupture, 8 (3.4%) had cerebral vascular spasm, and 4 (1.7%) had intraoperative brain edema. There were seven (3.0%) cases of postoperative infection, eight (3.4%) cases of postoperative cerebral infarction, one (0.4%) case of postoperative hematoma, and two (0.8%) patients had some form of cognitive impairment after surgery. Follow up after surgery demonstrated that 189 out of the 235 patients (80.4%) had favorable outcomes (mRS score 0–2), and 43 (18.3%) had poor outcomes (mRS from 3–5). There were three deaths (1.28%).

**Conclusions:** The keyhole approach has a quick postoperative recovery, a mild postoperative response, and a good surgical outcome. Our findings indicate that the keyhole approach is a safe and effective surgical method for the treatment of anterior circulation aneurysm.

## Introduction

Intracranial aneurysms (IAs) are very common and are the third leading cause of cerebrovascular disease (1). At present, the pathogenesis of this disease is not completely clear, but most studies believe that the occurrence of it is related to cerebral arteriosclerosis, vasculitis, and hypertension (2). IAs are the main cause of spontaneous subarachnoid hemorrhage (SAH), and about 85% of spontaneous subarachnoid hemorrhages are related to the rupture of an intracranial aneurysm (3). The incidence of aneurysmal subarachnoid hemorrhage (aSAH) worldwide is roughly 9/1,00,000 people per year, and its morbidity and mortality are both high. Approximately 85% of IAs are located in the anterior circulation of the circle of Willis. Therefore, timely treatment of patients is particularly important (4). For the treatment of patients with anterior circulation aneurysms, choosing an economical and effective surgical method is a problem faced by clinicians. They need to consider not only short-term efficacy but also the potential postoperative complications.

Wilson first proposed the concept of “keyhole surgery technique” in 1971 (5). The minimally invasive keyhole approach is a significant milestone in the development of surgical approaches for the treatment of IAs. After that, through years of practical demonstration, significant curative effects have been achieved in the treatment of anterior circulation aneurysms, effectively reducing the patient's treatment cycle (6, 7). The keyhole approach is often used to treat anterior circulation aneurysms, and the current common surgical methods are supraorbital keyhole approach (SKA) and pterional keyhole approach (PKA) (8). The safety, effectiveness, and minimal invasiveness of the keyhole surgical clipping treatment of intracranial aneurysms have been proved in clinical practice (9). Although the above-mentioned surgical method can promote patient recovery to some extent, there are still some patients with poor prognosis, permanent disability, and even death (10, 11). Therefore, in this study we reviewed the PKA and SKA treatment of IA and surgical techniques, and we compared the functional outcome and safety of the two keyhole approaches for intracranial aneurysm clipping.

## Materials and Methods

### Clinical Materials

**Figure 1:**
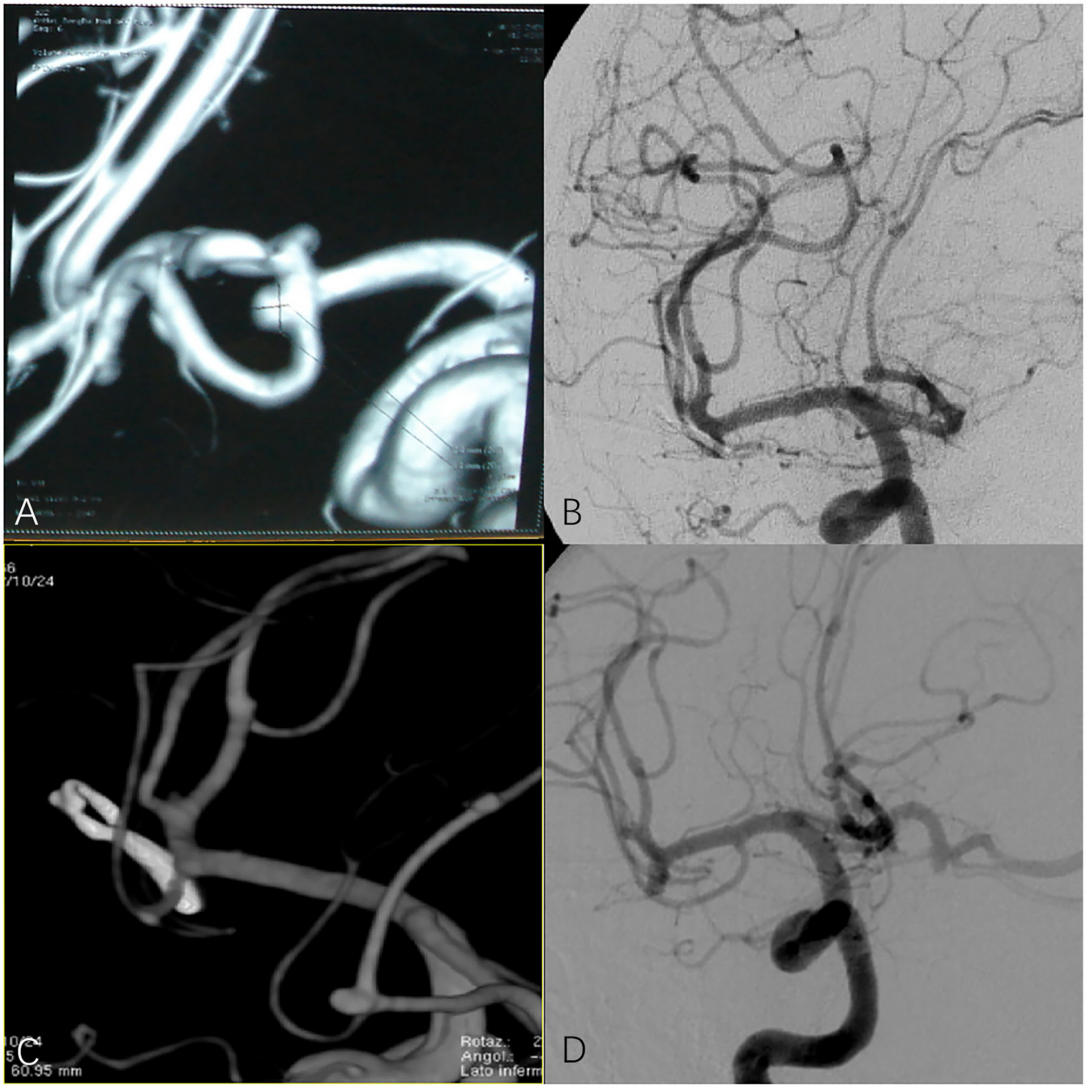
**(A,B)** Preoperative digital subtraction angiography (DSA) or computed tomography angiography (CTA) in patients with anterior communicating artery aneurysm. **(C,D)** CTA and DSA of patients with anterior communicating artery aneurysm after surgery.

We performed a retrospective study of 235 patients with 248 anterior circulation aneurysms who received SKA or PKA from the same surgeon in our hospital between January 2016 and January 2021. We excluded (1) patients with cerebrovascular malformation, including arteriovenous malformation (AVMs) and moyamoya disease (MMD), (2) patients with IA who were clamped at the same time during other operations, and (3) patients who were admitted to hospital with incomplete imaging and clinical data. The baseline characteristics of the 235 patients are shown in Table 1, including patient demographics, localization and size of the aneurysms, and Hunt-Hess grade. Preoperatively all patients were examined with digital subtraction angiography (DSA) or computed tomography angiography (CTA) to evaluate the angiographic architecture and brain condition (Figures 1A,B). After preoperative evaluation by the neurosurgeon, all selected patients were informed of the advantages and disadvantages of the choice of operative approach. After signing the informed consent and agreeing to the operation, SKA or PKA was performed, according to the clinical situation. If the patient has some form of cognitive impairment, this will be done by close relatives. Complications recorded include intracranial infection, postoperative hematoma, cerebral infarction, and cognitive impairment. Brain CT was performed immediately and at 1 month after discharge to rule out any. CTA was performed 2 weeks after the operation to check the clipped aneurysm and the presence of delayed bleeding or parenchymal changes. In some cases, DSA was performed when CTA was not considered appropriate for evaluating clipped aneurysms and parent arteries. When neurological problems occurred perioperatively, an appropriate imaging modality was an additional procedure (Figures 1C,D). Then CTA or DSA was repeated yearly. Patients were examined by a neurosurgeon at follow up, and those unable to return to the clinic were contacted by telephone (as part of routine care) to assess the recovery of the patients' neurological function and whether the aneurysm recurred or patients had rebleeding and so on. The patient's preoperative clinical condition was assessed according to the Hunt-Hess grading scale. The modified Rankin Scale (mRS) was used to measure long-term results during follow up. For mRS, a score of 0, 1, or 2 indicates a favorable result, and a score of 3 to 5 indicates a poor result.

**Table 1:** Baseline characteristics of patients.

Age, years	Min–max	24–85
	average	56
Male, no.	female	105 (44.7%)
	male	130 (55.3%)
Type of keyhole, no.	SKA	103 (43.8%)
	PKA	132 (56.2%)
Aneurysm location, no.	ACo.A	91 (36.7%)
	PCo.A	78 (31.5%)
	ACA	12 (4.8%)
	MCA	45 (18.1%)
	SHA	4 (1.6%)
	OA	15 (6.0%)
	AChA	3 (1.2%)
Aneurysm size, no.	>2.5 cm	14 (5.6%)
	1.5–2.5 cm	60 (24.2%)
	0.5–1.5 cm	136 (54.8%)
	<0.5 cm	38 (15.3%)
HUNT HESS grade, no.	I	46 (19.6%)
	II	94 (40.0%)
	III	79 (33.6%)
	IV	11(4.7%)
Unruptured aneurysm, no.		5 (2.1%)
Time to operation, no.	< 3 days	57 (24.3%)
	3–14 days	178 (75.7%)

### Statistical Analysis

The data are expressed as the average standard deviation of the normal distribution of continuous variables and the frequency or percentage of classified variables. All statistical analyses were performed with SPSS 23.0 (IBM Corp., Armonk, New York, USA). *p*-values < 0.05 were considered to indicate significance.

## Results

A total of 235 patients with anterior circulation aneurysm underwent complete SKA or PKA during the period of research (Table 1). In our cohort, 130 (55.3%) patients were male and 105 (44.7%) were female. The mean age was 56 years old (range 24 to 85). Of 235 patients, 230 (97.9%) had a ruptured aneurysm. SKA was performed in 103 patients (43.8%) and PKA was performed in 132 patients (56.2%). A total of seven locations were identified for 248 aneurysms in 235 patients, including 91 anterior communicating artery (ACoA; 36.7%), 78 posterior communicating artery (PCoA; 31.5%), 12 anterior cerebral artery (ACA; 4.8%), 45 middle cerebral artery (MCA; 18.1%), 4 superior hypophyseal artery (SHA; 1.7%), 15 ophthalmic artery (OA; 6.0%), and the anterior choroidal artery in three cases (AChA; 1.3%). All of the patients we studied who had ruptured aneurysms had SAH. For operative time, there were 178 (75.7%) patients who underwent surgery at 3–14 days. Using the Hunt-Hess grading scale, 46 (19.6%) patients were grade I, 94 (40%) were grade II, 79 (33.6%) patients were grade III, and 11 (4.7%) were grade IV.

Surgery-related details, intraoperative and postoperative complications, and hospital length of stay are shown in Table 2. For intraoperative complications, 31 (13.2%) had intraoperative aneurysm rupture, 8 (3.4%) had cerebral vascular spasm, and 4 (1.7%) had intraoperative brain edema. In terms of postoperative complications, there were 7 (3.0%) cases of postoperative infection, 8 (3.4%) cases of postoperative cerebral infarction, 1 (0.4%) with postoperative hematoma, and 2 (0.8%) patients had some form of cognitive impairment after surgery. The modified Rankin Scale (mRS) was used to measure long-term results during follow up. Follow up after surgery demonstrated 189 out of the 235 patients (80.4%) had a favorable outcome (mRS score 0–2), and 43 (18.3%) had a poor outcome (mRS score 3–5). There were three deaths (1.28%) (Table 3). There was no bleeding or recurrence in any patients during follow up.

**Table 2:** Surgery-related details, intraoperative and postoperative complications.

Operative time, mean ±SD, minutes		148 ± 47
Intraoperative blood loss, mean ± SD, ml		201 ± 98
Postoperative hospitalization, days	Min-max	7–15
	Average	9
Intraoperative complications, no.	Intraoperative aneurysm	31 (13.2%)
	rupture	
	Cerebral vascular spasm	8 (3.4%)
	Cerebral edema	4 (1.7%)
Postoperative complications, no.	Intracranial infection	7 (3.0%)
	Cerebral infarction	8 (3.4%)
	Hematoma	1 (0.4%)
	Mental symptoms	2 (0.8%)

**Table 3:** The modified Rankin Scale (mRS) of the postoperative patients.

Postoperative mRS	0–2	18 (80.4%)
	3–5	43 (18.3%)
	6	3 (1.28%)

## Discussion

In recent years, with the development of minimally invasive neurosurgery, the choice of surgical approach is not limited only to ensuring adequate exposure but also more attention is being paid to reducing intraoperative trauma and avoiding unnecessary neurological damage. Therefore, surgical treatment of anterior circulation aneurysms has gradually been transformed from a traditional transcranial approach to a minimally invasive keyhole surgery approach (12–14).

A significant advantage of the minimally invasive keyhole surgery approach is that the reduction of the bone window reduces the disturbance of normal intracranial nerves and blood vessels. This surgical approach uses the natural sulcus fissure and cistern as the surgical channel. It achieves the largest intraoperative visual field exposure with the smallest bone window design and allows precise individualized design before surgery. “Not only does it allow good exposure for aneurysmal clipping, but it also minimizes the incidence of surgical complications (15). With the development of neurointerventional materials and technologies, endovascular therapy has become the main trend in the treatment of IAs. However, compared with endovascular therapy, keyhole approach surgery has a wider range of indications. Surgical clipping is preferred for patients with internal carotid artery bifurcation aneurysm, MCA bifurcation aneurysms, aneurysm with a branching vessel, or aneurysm rupture combined with hematoma (11). At the same time, ISAT also indicated that the recurrence rate and rebleeding rate of endovascular therapy were higher than that in the surgical clipping group.

### Choice of Surgical Approach

The pterional approach was first proposed and promoted by Yasargil et al. and it is widely used in the field of neurosurgery (16). It is undeniable that compared with other approaches, the exposure of this surgical approach is wider and traction is reduced, but too much exposure leads to the possibility of opening the sphenoid sinus, which will increase the operative time and blood loss and will also lead to leakage of cerebrospinal fluid and increase the risk of intracranial infection after surgery. Therefore, the pterional keyhole approach (also known as mini-pterional keyhole craniotomy) is derived from the pterional approach, which provides new concepts for the development of subsequent surgical approaches (17). Tang et al. have been exploring the pterional keyhole approach to treat anterior circulation aneurysms (18). They confirmed that the PKA can overcome a series of shortcomings of the traditional approach. Craniotomy in this approach is small, usually only about 3 cm in diameter, which can shorten the operative time and hospital stay. The incidence of intracranial infections and other complications also tends to decrease significantly. Our experience shows that although the surgical exposure effects of the two techniques are equivalent, PKA causes less tissue trauma, requires less bone removal and operative time, and produces better cosmetic results.

**Figure 2:**
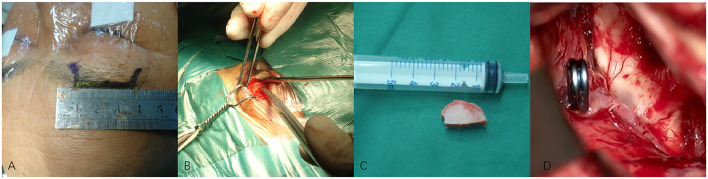
**(A–D)** Techniques during supraorbital keyhole approach (SKA) surgery in a patient with anterior communicating artery aneurysm (ACoA). **(A)** The cutting edge of upper edge of eyebrow arch. **(B)** The bone window of SKA. **(C)** The size of the bone window. **(D)** The aneurysm was clipped under a microscope.

**Figure 3:**
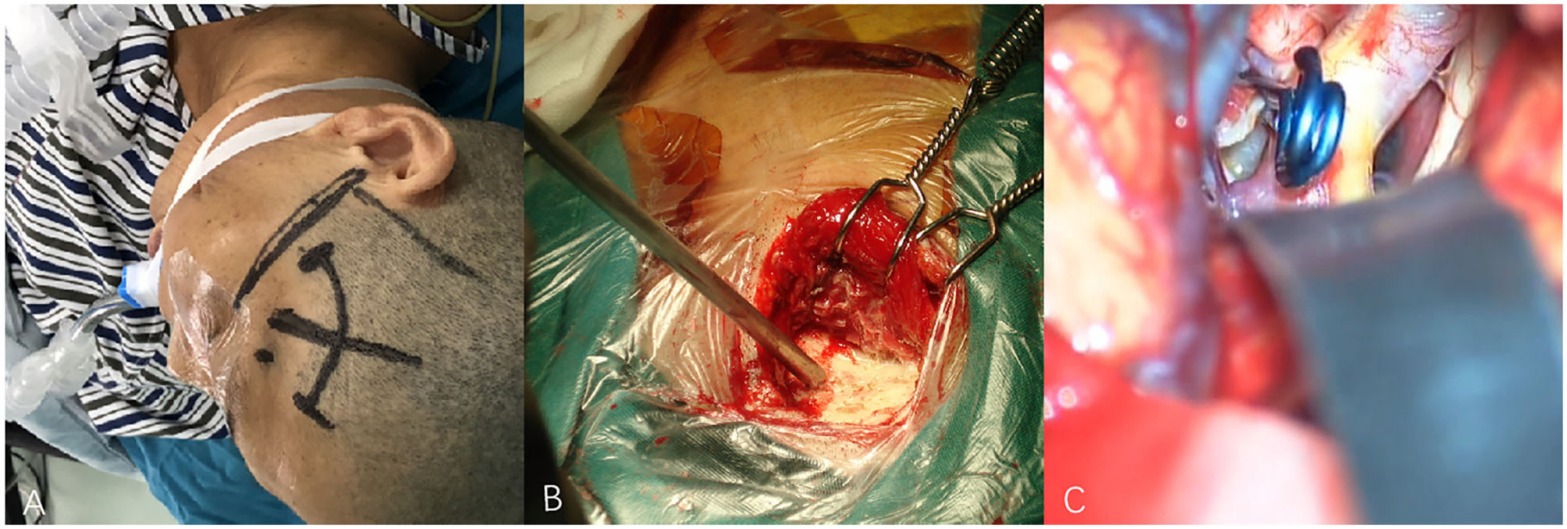
**(A–C)** Techniques during pterional keyhole approach (PKA) surgery in a patient with middle cerebral aneurysm (MCA). **(A)** The cutting edge of upper edge of pterion. **(B)** The bone window of PKA. **(C)** The aneurysm was clipped under microscope.

The unilateral subfrontal approach is a widely used traditional surgical method. It was first reported by Cushing in 1938 and is one of the most classic surgical methods. When the traditional subfrontal surgical approach is used, some tissues, nerves, and blood vessels around the lesion will be damaged (19, 20). The SKA is the result of optimized development on the basis of the traditional subfrontal approach. The advantage of this approach is that the temporal lobe does not need to be lifted, and the ipsilateral internal carotid artery, the proximal segments A1 and A2 of the anterior cerebral artery, the M1 and M2 segments of the MCA, the ACoA, and the posterior communicating artery can be reached when only the frontal lobe is lifted (21). Park et al. explored the method of the eyebrow keyhole approach to treat anterior circulation aneurysms and proved that the eyebrow keyhole approach has fewer complications and low mortality and verified that the keyhole clipping treatment is an effective and durable treatment method (22). In our experience, most patients after SKA surgery have excellent aesthetic results. Representative examples for SKA and PKA are shown in Figures 2, 3.

**Figure 4:**
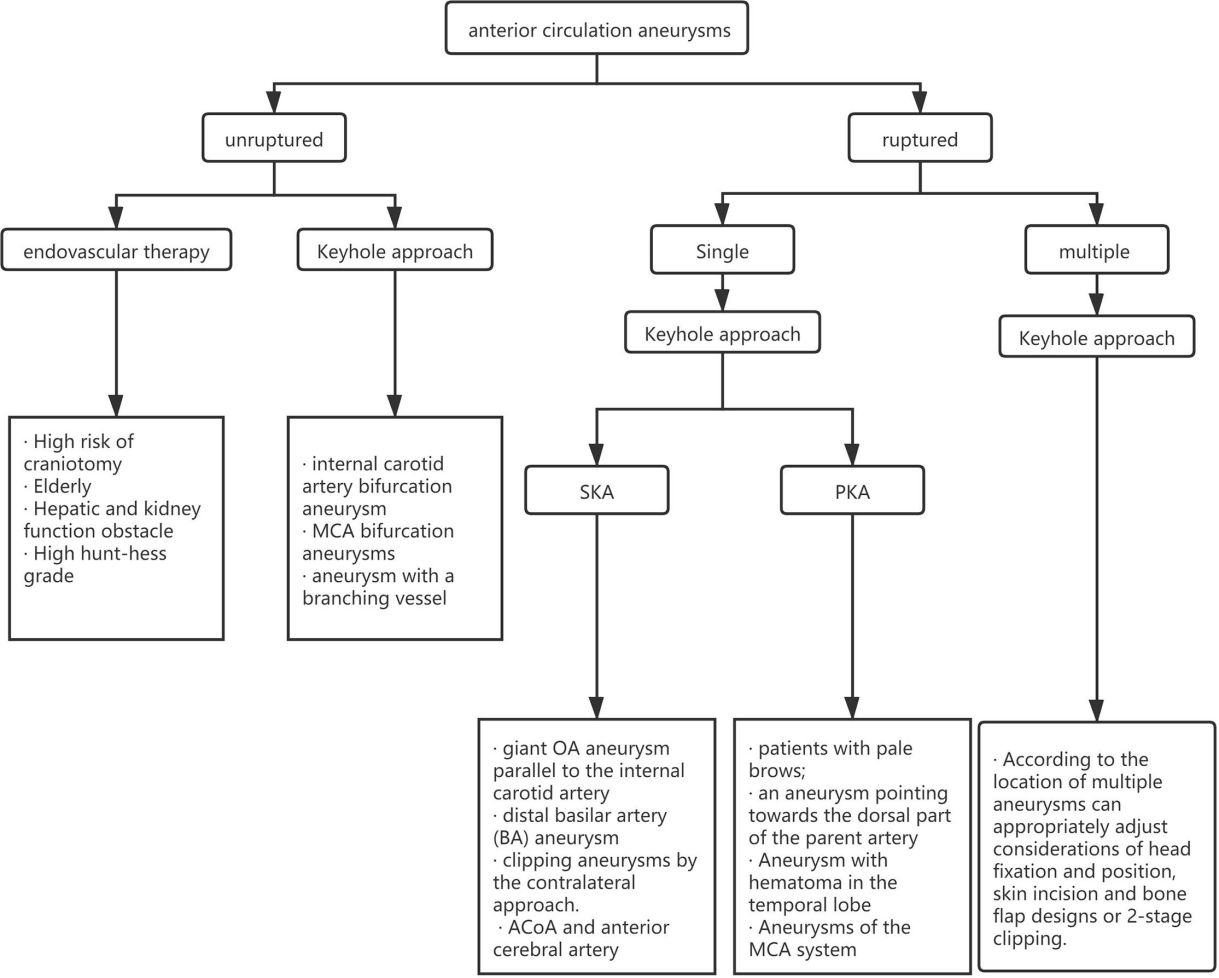
A clinical algorithm guiding clinicians to choose the best approach.

For the choice between PKA and SKA, Tra et al. and Wu et al. made a detailed comparison of the intraoperative conditions and postoperative complications of the two approaches (23, 24). They believe that the intraoperative and postoperative complications of aneurysm patients in the two surgical methods are not much different, and the prognosis for the two methods is equivalent at the time of discharge. Our experience shows that the two keyhole approaches have similar curative effects and fewer complications. However, we believe that the approach chosen by the surgeon should be closely related to the location of the aneurysm. We believe that the SKA is more suitable for aneurysms of the ACoA and anterior cerebral artery and that PKA is more suitable for aneurysms of the MCA system. The approach needs to be selected according to the condition of the patient, and the approach familiar to the surgeon is also one of the factors that need to be considered. We created a clinical algorithm to guide clinicians in choosing the best approach (Figure 4).

### Complications and Management

In a previous study, Chalouhi et al. compared the complications of the PKA and SKA in 87 cases of anterior circulation aneurysms. Surgical complications included six cases of intraoperative rupture (6.9%), two cases of epidural hematoma (2.3%), two cases of infection (2.3%), and eight cases (9.2%) of cerebral ischemia (including one case of clinical asymptomatic infarction). Recently, Lan et al. also performed a comparison of 195 SKA and 123 PKA procedures. There were 8 (2.5%) patients with symptomatic cerebral vasospasm, 5 (2.1%) patients had visual deterioration, 8 (2.5%) patients had intracranial vasospasm, and 26 patients (7.1%) had intraoperative rupture (25, 26). There are complications of the keyhole approach that need to be treated, but currently complications are relatively rare. Our research provides some treatment methods for intraoperative complications.

In our study, 31 aneurysms (13.2%) were ruptured during surgery. We found that aneurysm rupture was closely related to operation time and aneurysm shape and size. Our experience has found that the probability of intraoperative rupture in patients undergoing surgery within 1 week after diagnosis is significantly reduced. Blood blister-like aneurysms or irregular aneurysms and small aneurysms are relatively easy to rupture, but we have found that large aneurysms are not easily ruptured. The neck of the aneurysm is narrow and is easy to clamp, and rupture is relatively rare. At the same time, common causes of aneurysm rupture are fast intraoperative cerebrospinal fluid release, excessive traction of the aneurysm neck, and insufficient arachnoid separation. Most of the ruptures arise from the dome of the aneurysm and rarely on the neck of the aneurysm, and they have extensive bleeding and local hematomas. Once the rupture is found, one or more high-flow suction apparatus should be used immediately to remove the blood accumulation. After the operative area is clear, proximal parent should be temporarily clipped if possible. If not, the gelatin sponge should be wrapped and reinforced and clipping tried again. If clipping is ineffective after this, vascular rheosis should be considered to isolate the aneurysm from the responsible artery. Heparin reversal should also be considered if bleeding is associated with vascular perforation. We recommend (1) that in the preoperative period, the neurosurgeon should carefully examine all imaging studies (CTA and DSA) to be fully aware of the location of the aneurysm and the direction of the aneurysm dome. This both facilitates access to the aneurysm and reduces the probability of intraoperative rupture. (2) If preoperative evaluation shows there are high-risk factors for bleeding, the intracranial pressure should be fully reduced before treating the aneurysm. Glycol and high seepage salt water are commonly used osmotic diuretics. (3) Arachnoid dissection should be sharp and meticulous. (4) The Sylvian fissure should entirely be opened. (5) The neck of the aneurysm should be divided first, trying to avoid the top of the aneurysm without pre-clamping or clipping the aneurysm. (6) Extreme traction should be avoided. (7) Cerebrospinal fluid should be released slowly and if necessary, an external ventricular drain (EVD) and external lumbar drainage (ELD) can be used. (8) If the dissection of the aneurysm appears to be risky, temporary clipping should definitely be used. We use temporary clips quite frequently, especially in ruptured aneurysms. The duration of temporary clamping should be minimized. For older patients with atherosclerosis, the use of temporary blocking is more conservative. Bent temporary clips may be more suitable for proximal vascular control, while straight temporary clips are more suitable for distal vascular control. (9) Once uncontrolled bleeding occurs, brief cardiac arrest is induced by intravenous adenosine, which can cause hypotension and can facilitate rapid separation and temporary clipping. (10) Intraoperative intracranial protection, electrophysiological monitoring, Doppler ultrasound and other monitoring techniques can help reduce the risk of postoperative complications, while intraoperative fluorography and combined surgery can help reduce the risk of postoperative residual aneurysm or parent artery stenosis to improve the safety of surgery.

We found four (1.7%) patients with intraoperative cerebral edema. IA patients have pathological changes of increased intracranial pressure. Under the hypertension, increased intracranial pressure will reduce cerebral perfusion pressure and aggravate brain tissue metabolic disorders. The higher the Hunt-Hess grade is, the higher the intracranial pressure is, and the more difficult the treatment is. Therefore, intracranial pressure monitoring should be carried out in patients with high-level ruptured Intracranial aneurysm. If necessary, the intracranial pressure should be lowered before the operation. We commonly used osmotic diuretics such as glycol and high seepage salt water. During the operation, EVD and ELD can be used to release the cisternal cerebrospinal fluid to quickly lower the intracranial pressure and relieve cerebral edema. Decompressive craniectomy may be performed in patients with severe intracranial hypertension and brain swelling after aneurysm clipping.

Intraoperative vasospasm is also a relatively common intraoperative complication. It occurred in five (2.1%) of our patients. In general, papaverine solution (50 ml normal saline with 30 mg papaverine added) was used to repeatedly flush the operative field and basal cistern. This papaverine solution should be used to repeatedly rinse the surgical field and basal cistern the exposed arteries covered with a gelatin sponge impregnated with papaverine. A stock solution of nimodipine diluent (normal saline V/V = 1:10) should be warmed to the same temperature as the blood, then the exposed blood vessels in the operative area soaked in it for more than 10 min until the patient's blood pressure has increased. In the present study, although antispasmodic therapies were administered postoperatively, eight patients experienced symptomatic cerebral vascular spasms. In follow up, three patients had gradually recovered, one patient still had severe disability, and three of our patients died because of this.

Some special circumstances may be encountered, for example: (1) If the frontal sinus was breached during the operation, the mucosa in the frontal sinus can be completely removed, and the sinus cavity can be sealed with gentamicin-soaked gelatin sponge and bone wax. (2) For patients with difficulty in revealing aneurysms, partial gyrus rectus can be resected, but cognitive dysfunction, especially memory impairment, may occur after resection (25). Two (0.8%) patients were noted to have some form of cognitive impairment after surgery. We used cholinesterase inhibitors such as donepezil and carboplatin, which had a good effect in improving cognitive dysfunction, and both patients had a good recovery during follow up. (3) If the patient's condition is complicated by hydrocephalus, fenestration of the lamina terminalis can be carried out. However, a systematic review showed that fenestration of lamina terminalis did not reduce the risk of chronic shunt-dependent hydrocephalus (27), but one study found that tandem fenestration of the lamina terminalis and membrane of Liliequist can reduce the rates of shunt dependency (28).

The three deaths (3.09%) were all in patients with aneurysms in middle cerebral arteries. All three patients had vasospasm during the operation and a large area of cerebral infarction after the operation. Although we actively controlled vasospasm during the operation, lowered the intracranial pressure, and treated symptomatically after surgery, the three patients died within 3 days after the operation. Bulters et al. conducted a study on 200 clipped aneurysms and said the majority of poor outcomes are due to surgical complications, and most of these are vascular. They also think careful preservation of perforators and accurate clip placement remain the key factors in determining outcome in surgically treated good-grade SAH (29). Our experience shows that to reduce poor prognosis and postoperative death, correct and standardized surgical methods and timely and correct treatment of intraoperative and postoperative complications are the most important.

### Limitations and Generalizability

Because of the small bone window, small exposure range, and limited operating space, the keyhole approach requires the surgeon be familiar with the anatomy of the area, have extensive surgical experience, and the ability to deal with sudden aneurysm rupture and hemorrhage. The keyhole approach technique does not show the entire surgical field, if certain local anatomy cannot be fully understood, and some important blood vessels and structures around the aneurysm may be damaged (30, 31).

Finally, the limitations of this study are mainly related to the retrospective design, lack of randomization, and the surgeon's assessment of the results. Our results present the experience at a single center with specific surgical techniques and protocols. Our approach cannot find aneurysms in the posterior circulation and other locations. Since this is a retrospective study, further prospective studies are needed to determine the best surgical method in this situation.

## Conclusion

The keyhole technique is the hallmark of minimally invasive neurosurgery. The surgical skin incision is small, maintaining the patient's appearance, and the craniotomy is small, reducing the exposure and interference of normal brain tissue. The keyhole approach uses the normal intracranial anatomical space to reduce the brain retraction The operative damage is less, the postoperative complications (epilepsy, bleeding, etc.) are reduced, the blood needed for the operation is reduced, the postoperative recovery is quick, the cost is less, the postoperative response is mild, and the operative outcome is good. However, the keyhole approach currently has certain limitations and requires the surgeon to have the ability to deal with emergency complications during and after surgery. In the future, further improvements in surgical instruments, computer simulations, advanced microscopes, etc. may further improve the safety of this surgery.

## Data Availability Statement

The original contributions presented in the study are included in the article/supplementary material, further inquiries can be directed to the corresponding author.

## Ethics Statement

The studies involving human participants were reviewed and approved by EC of the First Affiliated Hospital of Bengbu Medical College. The patients/participants provided their written informed consent to participate in this study. Written informed consent was obtained from the individual(s) for the publication of any potentially identifiable images or data included in this article.

## Author Contributions

DS, YL, and ZJ designed the study. DS, XC, and ZS analyzed the data. DS, YL, and XZ wrote the manuscript. ZJ revised the manuscript and supervised the study. All authors approved the final version of the manuscript for publication.

## Funding

This study was supported by grants from the Natural Science Foundation of Anhui Province (No. KJ2018A0995).

## Conflict of Interest

The authors declare that the research was conducted in the absence of any commercial or financial relationships that could be construed as a potential conflict of interest.

## Publisher's Note

All claims expressed in this article are solely those of the authors and do not necessarily represent those of their affiliated organizations, or those of the publisher, the editors and the reviewers. Any product that may be evaluated in this article, or claim that may be made by its manufacturer, is not guaranteed or endorsed by the publisher.
